# The Impact of Plasma Membrane Ion Channels on Bone Remodeling in Response to Mechanical Stress, Oxidative Imbalance, and Acidosis

**DOI:** 10.3390/antiox12030689

**Published:** 2023-03-10

**Authors:** Martina Perin, Giorgia Chinigò, Tullio Genova, Federico Mussano, Luca Munaron

**Affiliations:** 1 Department of Life Sciences and System Biology, University of Torino, Via Accademia Albertina 13, 10123 Torino, Italy; 2 C.I.R. Dental School, Department of Surgical Sciences, University of Torino, Via Nizza 230, 10126 Torino, Italy

**Keywords:** bone remodeling, calcium signaling, ion channels, osteogenesis, mechano-sensing, acidosis, aging, physical activity, oxidative stress, Piezo

## Abstract

The extracellular milieu is a rich source of different stimuli and stressors. Some of them depend on the chemical–physical features of the matrix, while others may come from the ‘outer’ environment, as in the case of mechanical loading applied on the bones. In addition to these forces, a plethora of chemical signals drives cell physiology and fate, possibly leading to dysfunctions when the homeostasis is disrupted. This variety of stimuli triggers different responses among the tissues: bones represent a particular milieu in which a fragile balance between mechanical and metabolic demands should be tuned and maintained by the concerted activity of cell biomolecules located at the interface between external and internal environments. Plasma membrane ion channels can be viewed as multifunctional protein machines that act as rapid and selective dual-nature hubs, sensors, and transducers. Here we focus on some multisensory ion channels (belonging to Piezo, TRP, ASIC/EnaC, P2XR, Connexin, and Pannexin families) actually or potentially playing a significant role in bone adaptation to three main stressors, mechanical forces, oxidative stress, and acidosis, through their effects on bone cells including mesenchymal stem cells, osteoblasts, osteoclasts, and osteocytes. Ion channel-mediated bone remodeling occurs in physiological processes, aging, and human diseases such as osteoporosis, cancer, and traumatic events.

## 1. Introduction

Bone tissue physiologically undergoes a remodeling which is the result of a dynamic balance between bone formation by osteoblasts (OB) and resorption by osteoclasts (OC). These antagonistic processes are essential to support body weight and maintain its multiple physiological activities. Moreover, bone formation and resorption allow for response to dynamic mechanical forces and also assist in functional and metabolic demands. Nevertheless, in some specific conditions, homeostasis at rest can be altered by physiological stimuli as observed during exercise and load, but also associated with pathologies such as aging-related osteoporosis, metabolic diseases, cancer, and therapeutic interventions [1,2].

In particular, three stressors seem to prevail in bone remodeling: mechanical stress, oxidative imbalance, and acidosis. The identification of molecular sensors, transducers, and signaling pathways involved in these processes is of paramount importance in order to design more efficient clinical, pharmacological, and nutritional strategies, as well as to improve wellness through a healthy lifestyle and motor activity. Indeed, the promotion of physical exercise during growth increases the chances of strengthening bone and delaying the osteoporotic insurgence associated with higher bone mineral density and lower fracture incidence, with beneficial effects maintained in adulthood [3,4,5,6,7]. Moreover, exercise duration, type, and intensity drive exercise-induced inflammatory and metabolic responses that concur to improve bone remodeling [8].

A number of cellular proteins sense, transduce, and integrate environmental signals; these molecular machineries include, among others, plasma membrane ion channels. They are differentially expressed in great variety and redundancy in all bone cell types that include OB, OC, osteocytes, and stem cells, but also vascular endothelial cells (EC) that critically contribute to bone homeostasis and remodeling in a complex paracrine network [9,10,11,12,13,14,15,16].

**Figure 1:**
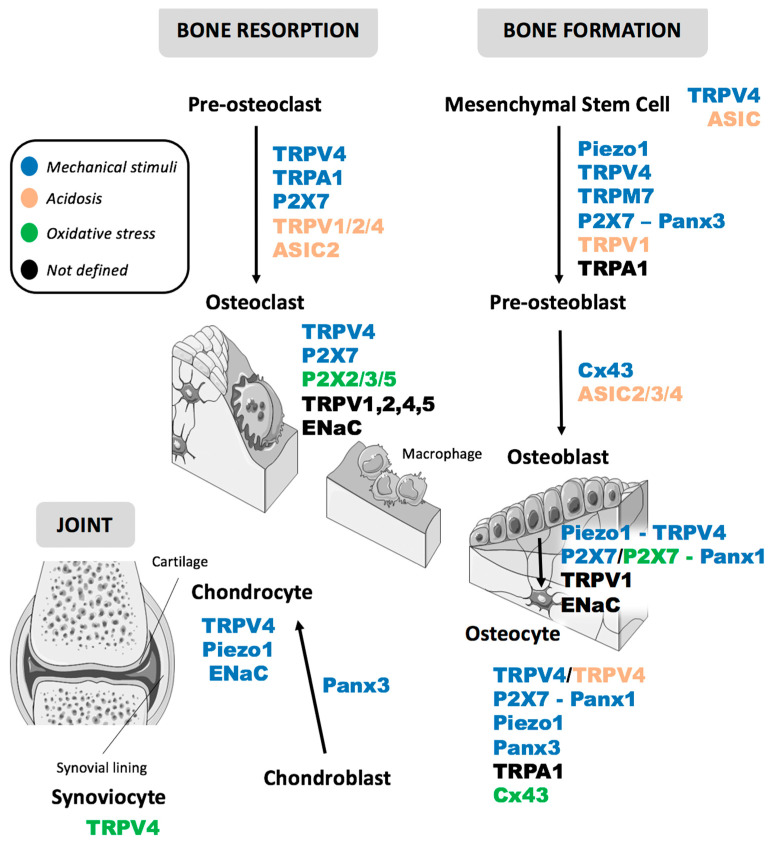
Schematic representation of the main plasma membrane channels involved in different phases of bone remodeling; the main stressors involved in their activation are represented by a color code as reported in the legend. The Figure was partly generated using Servier Medical Art, provided by Servier, licensed under a Creative Commons Attribution 3.0 unported license.

The first part of this review describes the characteristics of each stress and the second focuses on the most promising responsive candidates among the great variety of ion channels. In particular, we selected some members from Piezo, Transient Receptor Potential (TRP), Acid-sensing ion channels (ASIC), Epithelial sodium channels (EnaC), Purinergic receptors P2X, Connexins and Pannexins families involved in the different phases of bone remodeling and their susceptibility to mechanical stimuli, acidosis, and oxidative stress (OS) (Table 1 and Figure 1).

In order to dissect the specific intracellular responses triggered by each stress, in vitro experimental protocols are usually designed to apply single stimulations separately. However, in most pathophysiological settings, multiple environmental factors concur in an integrated pattern where different cell signaling pathways may interact; in this light, we discuss more appropriate strategies reported in the recent literature to accomplish the complexity of bone adaptation to changing multifactorial exposure through the convergence of ion channels.

## 2. The Role of Mechanical Stress, Oxidative Imbalance, and Acidosis in Bone Remodeling

### 2.1. Mechanical Stress

Bone load is necessary to maintain and control bone mass, growth, and remodeling, as originally noted by Galileo, later confirmed by Wolff’s law (1892), and further stated by Harold Frost in the 1980s ‘mechanostat’ concept. The formation, resorption, and adaptation of the skeletal system depend on the forces applied: in fact, the bone is weakened in their absence, as occurs in disuse osteoporosis which increases the incidence of fracture [83]. We currently know that mechanical stimuli tune various cellular functions, such as gene expression, protein synthesis, cell proliferation and differentiation. In an effort to develop new approaches for skeletal rejuvenation, the intricate intracellular pathways involved in the sensing and transduction of mechanical stresses are attracting the interest of biologists and clinicians [84].

The cellular components of bones include osteoblasts (OB), osteoclasts (OC), osteocytes, as well as their progenitor stem cells; all of them display different sensitivity to shear stress, hydrostatic pressure, mechanical stretch, and tension, together with matrix stiffness and alignment [84].

The most abundant population is represented by osteocytes, which respond to mechanical stimuli and propagate information to other cells in autocrine and paracrine modes [83]. During mechanical loading, osteocytes sense fluid flow (FF) in the lacunar-canalicular system while bone marrow mesenchymal stem cells (BMSC) in the medullary cavity [84]: these events drive BMSC proliferation and differentiation, altering their morphology, volume, and cytoskeleton arrangement and enhancing osteogenic gene expression. In the same cell type, oscillatory FF (pressure 1–5 Pa, frequency 0.5–2 Hz, duration 1–4 h) triggers the recruitment of differential gene patterns according to the intensity, frequency, and duration of shear stress. In particular, in the early stages of osteogenesis, short-term stimulation upregulates osteogenic markers such as Cox2 (cycloxygenase), Opn (osteopontin, a component of the extracellular matrix ECM), and Runx2 (transcription factor), while long-term treatment enhances collagen and matrix formation in late phases [84,85].

In addition to the externally applied mechanical load, the intrinsic mechanical properties of ECM strongly affect all cell bone types. For instance, ECM stiffness redirects MSC from adipogenic to osteogenic fate: the control of stem cell osteogenesis could be mediated by microtubule dynamics and deformation involving the microtubule-associated protein DCAMKL1 [84,86].

In summary, mechanical stresses exert multiple effects on bone cells: on one hand they promote morphological and cytoskeletal changes, on the other they alter tissue-specific gene expression.

### 2.2. Oxidative Imbalance

Oxidative stress (OS) is the imbalance of the oxidative state established by cellular pro- and anti-oxidant agents; it plays an important and widespread role in the physiopathology of many tissues as well as in senescence [87]. Aerobic cellular metabolism physiologically leads to the potentially threatening accumulation of reactive oxygen species (ROS) and free radicals (superoxide anion, hydrogen peroxide, hydroxyl radical) which are counteracted by antioxidants which act as scavengers and cell detoxifiers, dampening the overall damage. This is a common mechanism observed during aging and closely related to the development of osteoporosis. Indeed, upon persistent OS, a positive self-regenerating loop is produced leading to an array of pathophysiological changes [88]. Nonetheless, ROS are required for a wide range of healthy functions. In particular, they act as signaling molecules capable of modulating cell proliferation and differentiation, immune cell responses, and stem cell renewal [89]. In bone tissue, ROS physiologically enhance OC differentiation with the involvement of the RANKL pathway [90]. The redox state should be maintained in a precise balance to preserve the correct bone remodeling, since an increase in OC activity can easily lead to ROS accumulation and cellular stress. Indeed, ROS foster apoptosis of OB and osteocytes, thus promoting osteoclastogenesis and inhibiting mineralization and osteogenesis. The resulting redox dysregulation is involved in the pathogenesis of bone loss and can be observed, among the others, in osteoporosis.

Interestingly, mechanical and oxidative stresses are closely interconnected in bone: in fact, excessive mechanical forces affect not only bone mass and its microarchitecture, but also promote inflammation through the redox-sensitive NF-κB cascade [88,91]. The relevance of this pathway is supported by its role in the pathogenesis of osteoarthritis, and particularly in cartilage degradation [92]. 

Recently, age-related inflammation has been functionally coupled to metabolic changes observed during senescence. As an example, altered mitochondrial bioenergetic routes and increased glycolysis are reported in rat hepatocytes and aged skeletal muscle, where the responsiveness of mitochondrial oxidative phosphorylation to increase ATP demand is significantly reduced. Consistently, a glycolytic shift was detected in aging mice bone together with the activation of the Mitochondrial Permeability Transition Pore (MPTP) and tissue-specific mitochondrial dysfunction [93,94].

Based on these observations, growing attention has been focused on the promising use of antioxidants as valuable tools to counteract the predisposing factors of bone diseases (aging, osteoporosis, excessive loading) by the use of new pharmacological or nutritional approaches. 

Natural antioxidants include thiols, among which the most relevant in animals is glutathione (GSH, γ-glutamyl-cysteinyl-glycine), but also non-thiol compounds such as plant-derived polyphenols, vitamins—ascorbic acid, α-tocopherol, vitamins E and A, as well as enzymes such as ROS extruding catalases and GSH-consuming enzymes (mainly GSH-reductase and GSH-peroxidase).

Epidemiological studies suggest a link between nutritional intake of antioxidants and bone health through several mechanisms [95]. For example, long-term diets rich in polyphenols are reported to exert beneficial effects against harms related to chronic diseases including cancer, diabetes, inflammation, infection, neurological and cardiovascular disorders [96]. Polyphenols include phenolic acids, flavonoids, catechins, tannins, lignans, stilbenes, and anthocyanidins and are the most abundant dietary antioxidants found in fruits, vegetables, grains, legumes, chocolate, tea, coffee, and wine [97,98]. About 500 polyphenols are bioactive among the approximately 8000 known to date. Bone loss reduction has been correlated to the antioxidant activity of flavonoids, as evaluated in terms of bone resorption markers [99]. This protective aspect may be explained by several mechanisms that involve antioxidants-mediated anti-inflammatory efficacy, enhancement of osteoblastogenesis, suppression of osteoclastogenesis and osteo-immunological contribution [99].

Another line of evidence is provided by the use of resveratrol (Res), a polyphenolic compound mainly present in grape skin and seeds, that affects bone regeneration through different cellular pathways. For example, it mimics estrogen functions and potentiates Alkaline Phosphatase (ALP) activity along with OB differentiation through MAPK and estrogen receptor signaling. Res inhibits adipogenesis and promotes OB differentiation through Sirt activation and the restoration of Wnt/β-catenin pathway, while its anti-osteoclastogenic activity involves PGE2 inhibition and Sirt1-related anabolic signaling [100]. Accordingly, it regulates autophagy and promotes OB differentiation in a rat model of postmenopausal osteoporosis [101]. Finally, bone mineral density and ALP levels are increased upon Res treatment in clinical trials [102].

### 2.3. Acidosis

As already mentioned, the homeostatic imbalance between osteoclast-mediated resorption and OB-dependent bone formation is responsible for the insurgence of osteoporosis, an age-related skeletal disease characterized by decreased bone mass and altered architecture, leading to an increased risk of fragile fractures commonly found in the elderly. In addition to a high overall OS state, extracellular acidity is a paramount contributor to osteoporotic progression. The protons secreted by OC during bone resorption physiologically lead to the OC-bone interface acidification which interferes with bone cell biology. While an alkaline pH promotes mineralization and osteoblastic potential, acidosis in turn stimulates osteoclastic resorption. The OC sensitivity to protons involves early expression of the ovarian cancer G-protein-coupled receptor 1 (OGR1) which is responsive to H^+^ fluxes and is thought to play a role in osteoclastogenesis [103,104].

Acidic environment can also induce autophagy in OB [105] and inhibits OB-mediated biomineralization by interfering with ALP activity and impairing collagen proteins osteopontin and osteocalcin [106]. With aging, blood H^+^ levels increase and bicarbonate concentration is depressed, indicating progressive worsening low-level metabolic acidosis. Finally, it is worth noting that, as already noted, excessive skeletal loading is usually related to inflammatory processes and acidosis.

## 3. Ion Channels as Membrane Multi-Sensors and Transducers in Bone Remodeling: A Converging Machinery for Mechanical Stress, Extracellular Acidosis and Redox Balance

All forms of life require molecular machineries to detect and respond appropriately to a variety of environmental stressors. From a cellular point of view, several proteins are at the forefront of this communication between the intracellular and the external milieux: they include receptors, ion channels, and other biomolecules located in the plasma membrane.

In this review, we will focus on multisensory ion channels as peculiar sensor-transducer hubs, on which various mechanical and chemical stimuli converge to be encoded into specific intracellular signaling. In this context, the dual nature of channels accounts for a very selective and fast way to respond and adapt to stress changes. These ion-conducting pores are sensitive to multiple modulators of the pore gating and of its overall biophysical properties. For example, calcium-permeable ion channels display the special ability to rapidly trigger two types of high-content informational events: they mediate ionic currents across the plasma membrane thus tuning electrical voltage, and contribute to the regulation of cytosolic calcium concentration that is a chemical signal with universal biological relevance [107,108,109,110,111].

Starting from the analysis of the huge literature on the biophysical and physiological properties of ion channels and bone remodeling, we have chosen some candidates that show a multi-sensitivity to the three stressors previously described: mechanical stress, oxidative imbalance, and acidosis. Among them, proteins belonging to Piezo, TRP, ASIC, EnaC, P2XR, Connexin and Pannexin classes are likely to play a role in bone homeostasis and contribute to its adaptation scope to environmental changes. Indeed, calcium availability is critical to bone structural integrity, and intracellular calcium signals regulate many functions of osteoblast, osteoclasts and chondrocytes. The broad range of calcium-related proteins in MSC, well known for their ability to promote their osteoblastic differentiation, could support their involvement in OB precursors mechanosensitivity [112,113]. Consequently, their dysfunction is associated with bone formation and resorption disorders which are mediated by OB and OC, respectively [114].

Among the variety of ion channel gating modes, mechanical stretch is one of the most relevant for bone remodeling. The recruitment of mechanosensitive (MS) ion channels can occur upon application of forces to the lipid bilayer and lead to the conversion of the primary stimulus into electrical currents within milliseconds [115]. Piezo1 is directly gated by force-induced conformational changes [116], while other channels are indirectly mechano-operated through the mediation of associated membrane components such as the integrins and Connexin43, which will be discussed later.

### 3.1. Piezo

The mammalian Piezo family includes Piezo1 and 2 proteins which arrange in trimers acting as MS calcium-permeable channels. The physiological activation of Piezo follows the physical deformation of the lipid bilayer resulting from a finely tuned modulation by intracellular tethers of the cytoskeleton and by extracellular matrix [83,115,117,118,119]. This event can be mimicked in artificial lipid bilayers as well as by pharmacological treatments [116].

Although the mechano-gating is the most attractive feature of Piezo, they can also act as sensors for other biologically relevant variables, as revealed by patch clamp evidence reporting their inhibition by extracellular acidification below pH 6.9 [21]. In this review, we will mainly focus on Piezo1 since its contribution to mechano-transduction is well described [120]. However, some studies report Piezo2 involvement in MS in combination with Piezo1 [121].

Piezo1 is required for mammalian embryonic development and is mainly found in hollow organs, such as lungs, blood vessels, bladder, and gastrointestinal tract, but its recruitment is also reported in OB, osteocytes and chondrocytes thereby regulating osteogenesis and cartilage degradation in joints [18,122]. Similarly, Piezo1 regulates MSC fate thereby triggering osteoblastic differentiation and hindering the adipogenic one [17].

As aforementioned, the most relevant stressor modulating Piezo1 is the mechanical force (hence the name), which is a key factor in bone remodeling. In order to investigate its downstream intracellular targets, Piezo1 was activated with its specific agonist Yoda1 in MSC leading to the identification of ERK1/2 and p38 signaling as key pathway in correlation with Bone Morphogenetic Protein 2 (BMP2), a critical osteogenic growth factor [17]. The same effect can be obtained upon hydrostatic pressure application [17,123]. However, additional Piezo1-related signaling pathways are reported; for example, impaired Piezo1/2 expression in bone associates in vivo with decreased levels of Wnt/β-catenin, key mediators of OB differentiation, as well as of Hippo/Yap1, well known for their role in mechano-transduction [19]. 

Another study in vivo unveiled a role of Piezo 1 in bone loss after mechanical unloading by the use of the hindlimb suspension (HS) model which consists of suspending mice hind limbs to analyze Piezo1 function in knockout (KO) and wildtype conditions. HS reduces bone strength and alters OB physiology in *wt* mice but this dysfunction is blunted in *Piezo1* KO animals, thus suggesting that the channel is a key player in mechanosensitivity and the subsequent tissue remodeling mainly by acting on OB [18]. 

Piezo1 is also detected in osteocytes, which are the most abundant bone cells, where mechano-transduction is mediated by Piezo1-Akt signaling and suppression of sclerostin, an important regulator of bone formation [124]. Accordingly, the ablation of this channel in KO mice compromises bone responses to mechanical forces [20]. 

In addition to its physiological functions, a contribution of Piezo1 has also been reported in some bone diseases. In particular, its downregulation is related to osteoblast dysfunction in osteoporotic patients, opening the possibility for new selective therapeutic approaches [18]. A quantitative computed tomography (μ-QCT) study performed in Piezo1-deficient mice of different ages revealed that the newborn mice show no differences with *wt* animals [125]. The lack of effect in neonatal age could be explained by the absence of mechanical stimuli activating the channel, again strenghtening the hypothesis for its involvement in bone tissue mechano-sensing and transduction [125]. 

Since MS channels often work as an operational unit in an integrated manner, it is helpful to look at their overall outcomes in bone tissue. For example, both Piezo 1 and 2, in combination with other ion channels including TRPV4 (see below) and calcium-activated potassium channels K_Ca_, contribute to adult stem cell differentiation [126] depending on the stiffness of the hydrogels on which MSC are seeded [23]. Piezo1 and TRPV4 interplay is also found in periodontal ligament cell mechanotransduction [127]. 

Further insights into this networking behaviour could potentially shed new light and prompt innovative tissue engineering approaches and therapies [126]. 

### 3.2. TRP

Transient Receptor Potential channels (TRP) are a class of cationic ion channels grouped into six subfamilies (TRPC, TRMPV, TRPM, TRPA, TRPL and TRPP) according to sequence homology. They are composed of six membrane-spanning helices that usually organize in functional homo- or heterotetramers and can be regulated or modulated by a number of stressors, including heat, pressure, tension, shear stress, oxidative stress and hypoxia. TRP are ubiquitously and redundantly distributed in healthy and altered human tissues where they play a large variety of functions [128]. 

Several TRP channels are detected in different human and murine OB-like cell lines. More specifically, TRPC1,3,4,6, TRPV2,4 and TRPM4,6,7,8 are found in OB [51]. Some TRP affect skeletal calcium homeostasis interfering with intestinal calcium absorption (TRPV6), renal calcium reabsorption (TRPV5), OC differentiation (TRPV1,2,4,5) and chondrocytes (TRPV4) [29]. Furthermore, TRPV4, TRPA1 and TRPM7 are present in MSC together with the entire TRPC group and may regulate stem cells physiology and differentiation [129]. TRP are associated to bone resorption and osteoblast functions with a direct impact on osteoblastic and osteoclastic marker genes collagen 1 (COL1), ALP, osteocalcin (OCN), osteopontin (OPN), bone sialoprotein, Runx2, Cox2, c-Fos, tartrate-resistant acid phosphatase (TRAP), OPG/RANKL and NFATc1 [27,29,130].

#### 3.2.1. TRPV4

TRPV4 is a non-selective polymodal calcium-permeable channel widely expressed throughout the body and recruited in both physiological and pathological events. In bone tissue, TRPV4 has been associated with OC and OC differentiation, pathogenesis of osteoporosis and other metabolic bone diseases [131]. Its pore gating may be triggered by mechanical stress, temperature, or hypoosmolarity.

TRPV4 exhibits mechano-sensitivity. However, whether it fulfills the criteria for true MS channels has been debated [115]. Several mechanisms may mediate its mechano-activation, involving Phospholipase A2 (PLA2) and Phospholipase C (PLC). Upon osmotic swelling or shear stress exposure, PLA2 binds membrane phospholipids and releases arachidonic acid (AA), the fatty acid precursor of eicosanoids that include cytochrome p450-related epoxyeicosatrienoic acid (EET). Both AA and EET act as TRPV4 modulators [132,133,134,135].

The mechano-sensitivity of TRPV4 is detected in many cell bone subtypes, possibly interacting with other molecular machineries. In mouse osteoblastic cells, hypo-osmotic stress increases intracellular calcium through TRPV4 and TRPM3 to regulate RANKL and NFATc1 expression [25].

An important role is played by the primary cilium, an antenna-like, not motile structure that extends from the surface of most mammalian cell types into the extracellular space, where TRPV4 preferentially localizes in MSC osteoblast precursors and in differentiated osteocytes [22,31].

Interestingly, in MSC TRPV4 is triggered upon fluid shear stress associated with cilium stretch among other mechanisms. Following its association with Piezo1/2 and K_Ca_, TRPV4 controls MSC differentiation and upregulates the osteogenic genes Cox2 and Opn [126]. Furthermore, its recruitment with the chemical agonist GSK101 enhances collagen and calcium deposition by the same cells [22]. Similarly, the previously cited study on the osteocytes showed that fluid flow stress triggers a TRPV4 (but not Piezo1-) dependent-calcium influx in the primary cilium [31].

Notably, a threshold amount of fluid shear stress affects the microtubule network leading to the release of ROS through the involvement of NADPH oxidase 2, which, in turn, promotes TRPV4 opening, Ca^2+^ influx, and CaMKII kinase stimulation, finally leading to a decrease in sclerostin, a secreted inhibitor of bone formation [30].

Chondrocytes produce and maintain the articular cartilage by sensing and responding to changing mechanical loads; TRPV4 and Piezo are key actors in these physiological events and mediate mechanical and inflammatory signals [136]. Interestingly, TRPV4-related Ca^2+^ signaling plays a central role in the response to low-strain (physiological) stimuli (3% and 8%), while Piezo2 is recruited at high-strain (traumatic) levels (18%) [34].

Mechanical stimulation of TRPV4 has also been associated with osteoporosis due to its impact on OC survival [27] and the higher osteoporotic fracture risk related to TRPV4 deficiency [137]. In particular, and similarly to what was previously discussed for OB [25], OC differentiation enhanced by the channel involves the calcium-calcineurin-transcription of NFATc1 signaling pathway which promotes osteoclastogenesis-related cFos and TRAP gene transcription [27].

Both Piezo1 and TRPV4 have been detected in OB and their co-expression suggests a MS capability [26]. Interestingly, however, these two channels are unable to respond separately, suggesting the requirement of a coupling mechanism. Furthermore, brief shear stress induced by fluid flow selectively activates Piezo1 (but not TRPV4) raising intracellular calcium levels and recruiting PLA2. This event in turn triggers the opening of TRPV4, which is responsible for a second phase of Ca^2+^ influx ultimately leading to pathological events [138]. Similarly, the combined role of TRPV4 and Piezo1 has been explored in chondrocytes where both sense and respond to mechanical stimuli [34]. However, their role is not interchangeable, since TRPV4 opens at Cyclic Tensile Strain (CTS) low magnitude, while Piezo1/2 respond mainly at higher CTS level [34].

TRPV4 has also been linked to osteoarthritis (OA), a bone disease characterized by a gradual deterioration of the cartilage that protects the ends of bones within joints. Chondrocytes treated mechanically or with the TRPV4 agonist GSK101 significantly potentiate matrix biosynthesis and anabolic-related components [139]. A hypotonic condition triggers ROS production through TRPV4 recruitment in synoviocytes [35]. Interestingly, tissue stiffening activates TRPV4 and increases M1 macrophages infiltration [33]. ROS enhance M1 synovial macrophages polarization during OA synovitis through the ROS/NLRP3 pathway, acting as key pro-inflammatory agents; this event is counteracted by pharmacological inhibition of TRPV4 by HC067047 [32]. The prominent role of TRPV4 in the onset of an inflammatory environment has raised the opportunity to consider this ion channel as a potential therapeutic target for OA prevention [32].

Together with TRPV1 and TRPV2, TRPV4 contribution is reported in the formation of large OC promoted by acidosis [28,37,38], which impairs bone formation by enhancing Ca^2+^ excretion and OC resorption [103]. As an example, increased OC formation was observed in mild acidosis environment upon TRPV4 activation by specific agonists 4-α PDD [39]. In addition, also TRPV1 might be a component of the acid-sensing machinery, selective antagonist suppressed osteoclast formation and activity [40]. Finally, TRPV2-mediated Ca^2+^ oscillations have been observed in RANKL-activated preosteoclasts [38].

Consistent with these findings, TRPV1 genetic ablation and pharmacological depression restore the activity of quiescent OC, further supporting the relevance of this channel and its potential use in the treatment of osteoporosis [140,141].

TRPV1,2 and 4 inhibit OB differentiation from MSC through the involvement of TNF-α and other inflammatory cytokines [24]. TRPV1 stimulation via low level laser irradiation (LLLI) promotes OB proliferation, as well as the increase in osteoblastic markers Runx2, Osx, Alp, and Opn expression [36]; consistently, the selective inhibitor capsazepine completely reverts this effect. Finally, Ca^2+^ currents carried by TRPV2 could be required to sustain both preosteoclastic differentiation and late stages of osteoclastogenesis [106].

#### 3.2.2. TRPA1

TRPA1 is the only member of the TRPA group. It is a polymodal channel sensitive to tissue damage, noxious cold, endogenous compounds released by oxidative reactions, as well as to pro-inflammatory peptide bradykinin via the PLC signaling [142]. It is distributed in a large variety of cell types often in association with TRPV1, especially in nervous tissues. Consistently, the combination of these two channels is considered relevant in nociception, but also in the inflammatory milieu [143]. 

The evidence of TRPA1 expression in bone tissue is controversial; no transcriptional levels have been detected in vitro in OB and OC cell lines, while it is identified in MSC OB precursors [43], in bone from a breast cancer pain mice model [46] in periodontal ligament cells [42] and odontoblast-like cells [44]. Due to its multimodal gating and peculiar sensitivity to oxidative stress and inflammation, this protein should deserve future investigation in bone biology.

TRPA1 has previously been proposed as a potential primary MS calcium channel within the mammalian sensory nervous system [144], although recent models suggest a major role for the transmembrane channel-like proteins (TMC1 and TMC2) and transmembrane proteins (TMEM). TRPA1 recruitment by membrane stress has been observed using chemical agents and hyperosmotic solutions [47]. In addition, new light on its controversial activity mechano-sensor has been shed by studies in artificial lipid bilayers where the thiol reducing agent TCEP is able to abolish TRPA1 activity, suggesting an intrinsic mechanosensitivity dependent on the redox state [47]. TRPA1 has also been found in periodontal ligament (hPDL) cells; upon mechanical stimulation, hPDL selectively upregulate the protein (but not other mechanoreceptors), possibly supporting its functional role: the downstream pathway involves the phosphorylation of MAPKs ERK1/2, p38 and JNK [42]. In addition, TRPA1-mechano-activation upregulates CCL2, a chemokine ligand involved in osteoclastogenesis, in periodontal ligament cells [41,42].

Odontoblast-like cells co-express TRPA1 and TRPV4 that is known for its sensitivity to mechanical stretch [44]. The involvement of both these channels follows hypotonicity-induced membrane elongation and involves p38 MAPK downstream pathway: interestingly, treatment with the pro-inflammatory peptide TNFα upregulates TRPA1 and downregulates TRPV4 [44].

TRPA1 is sensitive to external irritants, as clearly shown in nociceptive sensory neurons [145]. Many factors critically involved in OS, including ROS, alkenyl aldehydes, 15d-PGJ2 and hydrogen peroxide (H_2_O_2_) are good agonists for this ion channel. Intriguingly, the effects of OS-associated multifaceted environment on bone tissue may include additional channels: a possible role of TRPV1-4 and TRPC5, other channels sensitive to the redox potential, may be explored in the future [145]. TRPA1 is well characterized for its dependence on OS. Indeed, several lines of evidence link TRPA1 activity to the accumulation of endogenous compounds directly produced by oxidative reactions [146,147], as well as to the recruitment of pro-inflammatory factors [142,148].

As mentioned above, polyphenol resveratrol (Res), among the others, improve bone regeneration. Even if the identity of the underlying molecular machinery is unknown, a number of cell targets have been identified in other tissues that should deserve validation in bone cells. Indeed, Res recruits plasma membrane voltage-gated calcium channels and calcium ATPase, as well as intracellular calcium channels [100]. In prostate cancer-associated fibroblasts, Res triggers calcium signals and HGF/VEGF secretion through the activation of TRPA1 N-terminal [48].

#### 3.2.3. TRPM7

Another TRP channel involved in bone metabolism is TRPM7, whose deletion causes embryonic lethality in mice [149]. TRPM7 is a cation channel (Ca^2+^ and Mg^2+^ permeant) covalently linked to a protein kinase domain: it is ubiquitously distributed throughout the body acting as a regulator of Mg^2+^ homeostasis, motility, and proliferation. Odontoblasts, the dentin forming cells capable of sensing mechanical stimulation, express predominantly TRPM7 [52] among the numerous MS TRP (TRPA1, TRPC1, TRPC6, TRPV1, TRPV4). Based on a careful pharmacological approach, Won et al. demonstrated that TRPM7 is a fundamental mechano-sensor facilitating intracellular Ca^2+^ signaling in odontoblastic process [53]. The importance of TRPM7 in dental mineralized tissues has been further proved by its upregulation during amelogenesis in ameloblasts and odontoblasts, while TRPM7 kinase-inactive knock-in mutant mice are affected by reduced enamel mineralization and weakened enamel structure, albeit independently of ion channel function [150]. To assess the role of TRPM7 in bone development, the same group [50] generated rx1-Cre-dependent Trpm7 mesenchymal-deleted mice and observed shortened bones and impaired trabecular bone formation, pointing out a possible impairment of chondrogenesis. The authors concluded that TRPM7 is critical as a cation channel rather than as a kinase in bone development.

TRPM7 membrane translocation is enhanced by fluid shear stress in MSC. In particular, intermittent fluid shear stress (IFSS) regulates osteogenic commitment of MSC through TRPM7-Osterix axis [49]. The cell response to IFSS included the up-regulation of Osterix, but not Runx2, and the activation of p38 and Smad1/5 pathways [49]. Recent observations reported that low-intensity pulsed ultrasound/nanomechanical force generators enhance osteogenesis of BMSC through regulation of TRPM7, actin cytoskeleton, and intracellular calcium oscillations [49,54].

As shown in the present collection of examples, the family of TRP proteins collectively represents a multifaceted molecular array that improves the bone’s functional flexibility in response to variable stressors from the environment.

### 3.3. ASIC/ENaC

The acid-sensing ion channels (ASIC) and epithelial sodium channels (ENaC) are members of a family of proteins that play critical roles in mechanosensation, chemosensation, nociception, and regulation of blood volume and pressure [151]. They form hetero- or homotrimers with subunits that share a common structure with two transmembrane and a large extracellular loop.

#### 3.3.1. ASIC

Acid-sensing ion channels (ASIC) are a group of proton-gated cation-permeable channels that belong to the family of the degenerin Deg/EnaC group [55,115,152,153] and are found in adult BMSC-derived OB [56]. Culturing OB in media with increasing pH, reduced osteoblastogenesis was observed under acidic conditions concomitant with a progressive upregulation of ASIC2, ASIC3, and ASIC4 [55]. This pH-dependent pattern could support a role of ASIC in the pathogenesis of osteoporosis, which is characterized by a sharply acidic pH responsible for osteoblastogenesis impairment. ASIC2 is enhanced in human bone cells from osteoporotic vertebral fractures and depressed during osteoblast differentiation and mineralization [55].

Interestingly, although they are canonically recognized as pH sensors, ASIC1-4 also participate in the regulation of vertebrate mechano-sensitivity. Indeed, they are associated with nuclear contraction in primary human MSC and specific MS functions in non-bone tissues are correlated with functional ASIC1a, ASIC1b, ASIC2a, and ASIC3 [56]. For example, ASIC2 acts as a cardiovascular baroreceptor and is widely distributed in the somatosensory system [154,155], while ASIC3 is detected and functional in dorsal root ganglion neurons [156]. The potential biophysical mechanism explaining a direct ASIC mechano-sensing is not fully elucidated, and a role for etero-multimeric complexes or co-expression with true MS channels such as Piezo is under investigation [157].

Interestingly, ASIC are inhibited by resveratrol in male rat OS [158]. Res also interferes with ASIC3 expression thereby promoting cell autophagy and leading to an overall attenuation of bone cancer pain [57]. A direct involvement of ASIC in bone remodeling in response to mechanical or OS remains to be experimentally evaluated. 

#### 3.3.2. ENaC

The amiloride-sensitive epithelial sodium channel (ENaC) is a major contributor to intracellular sodium homeostasis; the αENaC subunit is found in skeletal cells including articular chondrocytes and OB where it has been proposed to contribute to mechanotransduction, sodium transport and extracellular sodium sensing. Interestingly, a correlation between bone Na^+^ content and bone disease has been reported, suggesting that ENaC-mediated Na^+^ regulation may influence osteogenesis [60,61,159].

UMR-106 OB-like cell line and human OB in primary culture express αENaC subunit [59]. Bovine α-ENaC exhibits a pressure-induced activation in lipid bilayers and when subjected to a hydrostatic pressure gradient [160]. Accordingly, reconstitution of rat a-ENaC in LM(TK2) cells, a null cell for stretch-activated, nonselective cation channels, provides a mechanically-gated channel permeable to sodium and calcium ions; elegant patch clamp experiments describe its biophysical properties upon application of negative pressure, cell swelling, or patch excision [59].

Osteoblastic proliferation, differentiation, and osteogenic gene expression is under the control of a cGMP/PKGII-dependent regulation of ENaC expression [58].

Intriguingly, ENaC displays multimodal gating not only sensitive to extracellular sodium and mechanical forces, but also to oxidative stress; EnaC upregulation mediates kidney cortex hypertension triggered by EGF, insulin, and IGF-1 via ROS production and subsequent depolymerization of the actin microfilaments [63]. A similar correlation between ENaC function or expression and ROS production in bone cells remains to be investigated.

Some evidence is provided for the EnaC function in OC obtained by differentiation of rat bone marrow cells. Exposure to different concentrations of amiloride significantly inhibited the expression of EnaC, reduced the number of osteoclasts as well as the number of bone resorption pits on bone slices and the level of OC-specific gene cathepsin K. ENaC contribution in the regulation of OC differentiation and bone resorption should deserve more detailed studies [62].

### 3.4. P2X Purinergic Receptors

P2X purinergic receptors (P2XR) are ligand-gated cationic channels primarily activated by extracellular ATP (eATP). The two-spanning transmembrane domain subunits associate into functional homo- or heterotrimers complexes structurally similar to ASIC/EnaC. P2XR are sensitive to different extracellular stimuli including biologically relevant inorganic ions, such as extracellular protons (P2XR share some structural similarities with ASIC), Zn^2+^ and Ca^2+^, that allosterically modulate channel activity [161,162,163,164]. Furthermore, hypoxia interferes with P2XR expression and membrane targeting both in physiological and pathological conditions [111].

Some P2XR are found in bone cells, and OC in particular show the highest variety of isoforms. P2X7R seems to be the prevalent member, contributing to normal and pathological bone metabolism with a key contribution to the early stages of bone repair; its deficiency leads to bone loss and susceptibility to fractures [70]. In addition, it is also involved in OB differentiation and osteogenesis [66,67,165]. P2X7R knockout results in impaired bone formation and lower mechano-sensitivity in vivo [165]. The molecular mechanism underlying mechano-sensitivity of P2X7 was explored in MSC subjected to physiological and supra-physiological mechanical loading; early expression of mechano-related genes such as c-Jun, c-Fos, and nuclear factor-κB was observed together with increased levels of eATP and the release of pro-osteoclastogenic factors; consequently, the overall OC number was increased [64].

P2X7R mechano-transduction not only mediates OC proliferation, but also the osteocyte mechano-sensitivity [45]. In these cells, estrogen depletion results in an inability to perceive mechanical stimuli that correlates with a change in the osteocyte mechanosomes, proteins complexes formed by P2X7R and Pannexin 1 (Panx1, see also below) [45,166].

Similarly, mechanical information is transduced through P2X7R in OB where shear stress recruits P2X7R and P2Y6R, thus promoting NF-kB translocation and degradation [65]. Since OC are another target for NF-kB regulation, a mechano-induced crosstalk between OB and OC seems likely [167].

P2X7R is the most abundant P2X form in OC and affects their differentiation [64]. It has been associated with osteoporotic progression through an active involvement in OB-to-OC signaling, where it supports OC survival. Consistently, its absence results in increased bone loss, due to a significant OB loss and OC increase, thus leading to an imbalance that explains the observed excessive bone resorption [68]. The potential causes of P2X7R loss have been investigated in some studies which have highlighted the occurrence of single nucleotide polymorphisms (SNP) possibly correlated with osteoporosis incidence; they can be distinguished between loss-of-function SNP that are associated with bone mineral density (BMD) decrease and gain-of-function SNP that reduce the risk of disease development [168,169]. Importantly, the number of functional variants identified in the P2X7R sequence is very high, suggesting its crucial role in the pathogenesis of osteoporosis.

Other P2XR members regulate bone resorption, especially by promoting a pro-inflammatory microenvironment [70]. P2X5R is expressed during OC maturation where it mediates inflammasome activation and IL1β release, which are required for OC-mediated bone loss [69]. Finally, osteoclastic P2XR2/3 mediate bone resorption and pain [70].

### 3.5. Connexins and Pannexins

Connexins (Cx) and Pannexins (Panx) share a similar structure with four transmembrane domain subunits arranged in functional examers [170]. The Panx family is relatively small and consists of three members (Panx-1-3) while Cx group includes more than 20 proteins [79].

Connexins assemble at the level of the plasma membrane in oligomeric complexes that play critical roles in many cellular processes including messenger transport, cell coupling, morphogenesis, differentiation, and growth in a wide variety of tissues. They are physiologically activated by mechanical stimulation, showing also sensitivity to pH and to the reduction of extracellular calcium concentration. In addition, Cx43 and Cx50 are also recruited by OS and other forms are regulated by phosphorylation [171,172,173].

Unlike Cx, Pannexins do not form cell–cell channels but act mainly as single-membrane channels [174]. However, they share some functions with the Cx family such as the involvement in Ca^2+^ signaling [175]. Of the three members of PanX family, Panx1 is by far the most studied. Its activity is mediated by several mechanisms including stretch, K^+^ and Ca^2+^ currents, phosphorylation and C-tail cleavage [77]. Mechanical activation of Panx1 triggers intracellular calcium waves and the extracellular release of ATP [176]; therefore, it is often associated with purinergic signaling involved in many processes such as apoptosis, blood pressure regulation, neuropathic pain, and excitotoxicity. Panx1 is also sensitive to changes in extracellular pH; alkaline environment induces a slight reduction in Panx1-mediated conductance, while a stronger effect is detected in terms of ethidium uptake (increase with alkalization and decrease with acidification) [77].

Some Cx and Panx are present in bones and modulate tissue remodeling, as well as osteoprogenitor and chondrocyte cell differentiation [79].

#### 3.5.1. Connexins

Bone cells are functionally connected in a sort of functional syncytium through the gap junction channels including Cx proteins [177]. Within this family, Cx43 is one of the most important members for its key roles in bone embryogenesis, cell differentiation and mineralization. Several lines of investigation lead to the conclusion that the non-junctional MS Cx43 hemichannels are essential during the early stages of OB differentiation and maturation, providing an efflux pathway for ATP or prostaglandin E2 (PGE2) [178,179,180,181]. Cx43 hemichannels are probably not directly mechanosensitive being their response possibly mediated by integrins, as suggested by the ability of fluid flow shear stress to promote the interaction between integrin α-5 and Cx43 and the following PGE2 release from osteocytes [71].

As already mentioned, both Cx43 and Cx50 are sensitive to OS particularly upon hydrogen peroxide stimulation [182] and a potential protective function is reported especially in osteocytes [72]. OS and connexins contribute to the pathogenesis of osteoporosis. In OS state osteocytes downregulate Cx43, while the opening of Cx43 hemichannels is protective against cell injury caused by oxidative imbalance [73]. Cx43 and Cx43-EGFP hemichannels are activated following an increase in the intracellular redox potential, possibly through cytoplasmic cysteine residues in the C terminus [183].

Finally, Cx26 and Cx43 hemichannels are affected by pH fluctuations; increasing extracellular pH from 7.4 to 8.5 significantly enhances Cx43-mediated Ca^2+^ current in HeLa cells [74]; the same potential feature could be investigated in bone cells. 

#### 3.5.2. Pannexins

Panx1 and 3 are involved in the integrity and function of the interconnected network of OB, osteocytes and OC [78,79,80,184,185].

The mechano-sensitivity of Panx1, together with its ability to release ATP, explains its relationship and interaction with P2X7R [186]. The formation of a functional complex among the two proteins has been described in osteocytes, where it could mediate apoptosis [75,76]. Panx1, P2X7R, and the T-type voltage-operated calcium channel CaV3.2-1 co-localize with integrin β3 on osteocyte processes, likely providing a specialized MS machinery that functions in a distinct manner [83], [187]. The complex is recruited following shear stress and osmotic pressure, while P2X7R mechano-transduction seems indirect and is likely mediated by Panx-1-dependent ATP release into the extracellular medium. The accumulated eATP then binds to the low-affinity P2X7R triggering the opening of its pore with an additional influx of calcium and potassium efflux [135,188]. Similar cooperation between ATP and P2X7R has been elucidated in other contexts such as inflammasome assembly and caspase-1 recruitment in monocytes and macrophages [189,190].

Although, as mentioned before, Cx43 hemichannels are proposed to mediate mechanically-induced ATP and PGE2 release in OB, Cx43 silencing fails to affect this processes [71]. In contrast, PGE2 production in response to fluid shear stress is abolished in P2R7R receptor-null OB and ATP-induced Yo-pro dye uptake (a marker of P2XR7 macropore activity) is attenuated following treatment with P2X7R or Panx1 channel blockers in wild type cells [71]. These data collectively indicate that Panx1 and P2X7R probably form a molecular complex involved in OB mechano-signalling [71]. 

Pannexin 3 (Panx3) is involved in the development and ageing of bone and cartilage as well as in osteoarthritis and intervertebral disc degeneration with protective or detrimental effects depending on the nature of the disease (age-related or injury-induced) [78,191,192]. The mechanism responsible for Panx3-mediated osteoblast differentiation from MSC is based on its multiple channel activities as hemichannel, endoplasmic reticulum (ER) Ca2+ channel, and gap junction. In particular, similarly to Panx1, its pore opening at the plasma membrane releases ATP into the extracellular environment leading to purinergic and PI3K/Akt signaling activation; these events could in turn mediate the opening of Panx3 localized on ER membranes and the subsequent Ca^2+^ release from intracellular stores. The downstream event is the NFATc1 translocation ultimately promoting osteogenesis-related genes Ocn and Alp, known to support OB differentiation [78].

The molecular mechanism of Panx3 involvement in chondrogenesis partially resembles that observed in osteogenesis; the inhibition of CREB signalling following Panx3 –induced release of ATP decreases chondrocytes proliferation and promotes their differentiation [81].

Although the role of Panx3 in bone remodeling has been established, its behavior in response to mechanical stretching, pH, and OS needs further investigation. OA pathogenesis is characterized by cartilage degradation, bone remodeling and synovial inflammation, where chondrocytes are important players [193]. Panx3 upregulation in OA and the protective effect of its deletion [193] are in agreement with the previously described role in chondrocytes differentiation. Mechanical stimuli applied to chondrocytes trigger ATP release [194]; although the precise contribution of Panx3 remains elusive, the presence of Runx2 binding sites in Panx3 promoter supports this possibility since Runx2 enhancement is related to mechanical loading [82,174].

## 4. Conclusions

Among the huge variety and redundancy of ion channel-forming proteins in bone tissue, some of them represent intriguing objects of investigation as they allow the major stressors in bone, mechanical loading, oxidative stress, and acidosis to converge and integrate their information content.

The search for molecular machineries that gain bone ability to translate the environmental stimuli into adaptive responses through remodelling of its own cellular components is of great interest for two main reasons. Firstly, it improves our current knowledge on physiological bone dynamics; secondly, it enables us to devise biomedical strategies and wellness behaviours that help to adequately treat or prevent damage from aging or acute and chronic diseases. 

## Figures and Tables

**Table 1:** List of the plasma membrane ion channels involved in bone remodeling and their role in physio-pathological processes; **↑**: increased, **↓**: decreased. OS: Oxidative Stress. MSC: Mesenchymal Stem Cells, CAF: Cancer Associated Fibroblasts, mpkCCDc14: mouse cortical collecting duct cells.

Protein	Mechano	pH	OS	Cell Types	Physiological Process	Ref.
Piezo1	X			MSC	↑ osteoblast differentiation	[17]
					↓ adipogenic differentiation	
					↓ osteoporosis	[18]
	X			osteoblast	↑ osteoblast differentiation	[19]
					mechanotransductive processes	
	X			osteocyte	↑ osteogenesis	[20]
					mechanotransductive processes	
				chondrocyte (in vivo mouse model)	↑ cartilage degradation	[19]
		X		HEK (transfected)		[21]
TRPV4	X			MSC	↑ osteogenesis	[22]
	X				mechanotransductive processes	[23]
		X			↓ osteoblast differentiation	[24]
	X			osteoblast	mechanotransductive processes	[25,26]
	X			osteoclast	↑ osteoclast survival	[27]
	X	X			↑ osteoclast differentiation	[28,29]
	X		X	osteocyte	↑ osteogenesis	[30]
	X				mechanotransductive processes	[31]
	X		X	chondrocyte	↑ macrophages infiltration	[32,33]
	X				mechanotransductive process	[34]
	X		X	synoviocyte		[32,35]
TRPV1	X	X		osteoblast	↑ osteoblast proliferation ↑ osteoblast differentiation	[36,37]
TRPV1 TRPV2 TRPV4		X		osteoclast	↑ osteoclast formation	[29,37,38,39,40]
TRPA1	X			osteoclast	↑ osteoclast formation	[41,42]
				MSC		[43]
	X			periodontal ligament cells	mechanotransduction	[41,42]
	X			odontoblast-like cell	inflammation	[44]
				bone cancer cell		[45,46]
	X			artificial bilayer	mechanotransduction	[47]
			X	prostate CAF		[48]
TRPM7	X			MSC	↑ osteoblast differentiation	[49]
					↑ chondrogenesis	[50]
				pre osteoblast	↑ osteoblastic differentiation	[51]
	X			odontoblast (in vivo mouse model)	mechanotransductive processes	[52,53]
	X			osteoblast		[49,54]
ASIC1-4		X		MSC	↑ osteoclastogenesis (ASIC2) ↓ osteoblastogenesis	[55]
	X			MSC	mechanotransductive processes	[56]
			X	bone cancer cell		[57]
ENaC				osteoblast	↑ osteoblast proliferation and differentiation	[58,59]
	X			chondrocyte	mechanotransductive processes	[60,61]
				osteoclast	↑ osteoclast differentiation	[62]
			X	mpkCCDc14		[63]
P2X7	X			MSC	mechanotransductive processes	[64]
	X			osteoblast		[65]
	X			osteocyte		[45]
	X			osteoblast	↑ osteoblast differentiation	[66,67]
	X			osteoclast	↑ osteoclast survival and differentiation	[64,68]
P2X2/3/5				osteoclast		[69,70]
Cx43	X			osteoblast	↑ osteoblast differentiation	[71]
			X	osteocyte		[72,73]
		X		HeLa		[74]
Panx1	X			osteoblast	mechanotransductive processes (in complex with P2X7)	[45,71,75,76]
				osteocyte		
		X		zebrafish retina (in vivo)		[77,78]
Panx3				osteoblast	↑ osteogenesis	[79,80]
				MSC	↑ osteoblast differentiation	
	X			osteoblast (MC3T3 and in vivo mouse model)	↑ osteoblastogenesis	[79,81,82]
	X	X		chondrocyte (in vivo mouse model)	↑ chondrogenesis	

## Data Availability

Not applicable.
